# A decade of endemic human coronaviruses: epidemiological patterns and clinical significance in an adult cohort at a tertiary care hospital in Germany

**DOI:** 10.1186/s12879-026-14377-1

**Published:** 2026-09-18

**Authors:** Christian Propach, Mario Hönemann, Vladan Vučinić, Armin Frille, Tom Berthold, Sandra Bergs, Melanie Maier, Corinna Pietsch

**Affiliations:** 1 Virology Department, Institute of Medical Microbiology and Virology, University of Leipzig Medical Center, Leipzig, Germany; 2 Medical Clinic for Hematology, Cell Therapy, Hemostaseology and Infectious Diseases, Comprehensive Cancer Center Central Germany, University of Leipzig Medical Center, Leipzig, Germany; 3 Fraunhofer Institute for Cell therapy and Immunology, Leipzig, Germany; 4 Division of Respiratory Medicine, Department of Medicine II, University of Leipzig Medical Center, Leipzig, Germany

**Keywords:** HCoV, Coronavirus, Epidemiology, Respiratory infections, Respiratory viruses

## Abstract

**Introduction:**

The four endemic human coronaviruses (HCoV-OC43, -NL63, -HKU1, and − 229E) are common respiratory pathogens. Studies on HCoV epidemiology in Germany are limited. Therefore, this study aimed to characterize the epidemiology and clinical significance of HCoV-infections in adult patients over 11 seasons at a tertiary care hospital in Germany.

**Methods:**

Over 29,000 respiratory specimens were analyzed using nucleic acid amplification assays for respiratory pathogens between 2012 and 2022. Additionally, clinical data were retrospectively retrieved from patient records for cases with confirmed HCoV infections.

**Results:**

During the study period, 469 unique adult cases were identified. HCoV-OC43 (38.6%) and HCoV-NL63 (25.6%) were most frequently detected, followed by HCoV-229E (22.4%) and HCoV-HKU1 (13.4%). HCoVs exhibited strong seasonality, with most detections occurring during the winter months, peaking between January and March. Sporadic detections of HCoV-OC43 and HCoV-NL63 were observed in summer. While HCoV-NL63 was detected year-round, HCoV-OC43 and HCoV-HKU1 displayed a biennial circulation pattern. No consistent pattern was observed for HCoV-229E. Across all HCoV cases HCoV-OC43 were more frequently associated with dyspnea (48.1%, *p* = 0.004), lower respiratory tract infection (45.3%; *p* = 0.018), exacerbation of obstructive lung disease (17.6%, *p* = 0.002), and systemic prednisolone administration (16%, *p* = 0.007). HCoV-HKU1 infection was more frequently detected in patients with lymphoma (35.6%, *p* = 0.002) and associated with higher in-hospital mortality (18.6%, *p* = 0.042). Overall, 119 co-infections (26.0%) were identified, including viral (10.9%) bacterial (10.5%), and fungal (1.7%) pathogens. Co-infections were significantly associated with LRTI (p < 0.001), need for mechanical ventilation (p = 0.001), and ICU admission (p < 0.001).

**Conclusion:**

Endemic coronaviruses exhibit complex circulation patterns characterized by distinct seasonality. The cyclic behavior of HCoV-OC43 and HCoV-HKU1 suggests possible immunological interactions within this virus family. The clinical presentation and severity of HCoV infections are highly dependent on the age group, comorbidities of the affected patients, and co-infections.

**Supplementary Information:**

The online version contains supplementary material available at https://doi.org/10.1186/s12879-026-14377-1.

## Introduction

Respiratory infections are among the most common human infections in both children and adults and impose a substantial financial burden on healthcare systems worldwide [1]. Several viral families, including coronaviruses (CoVs), are major causative agents of respiratory infections [2, 3].

Coronaviruses are classified into Alpha-, Beta-, Gamma- and Deltacoronavirus [4]. To date, seven coronaviruses have been identified as human pathogens. Whereas HCoV-NL63, HCoV-229E (both alphacoronaviruses), HCoV-OC43, and HCoV-HKU1 (both betacoronaviruses) are referred to as endemic or seasonal CoV [5]. SARS-CoV, MERS-CoV and SARS-CoV-2 (all betacoronaviruses) are associated with epidemic or pandemic potential. Studies conducted prior to the SARS-CoV-2 pandemic showed that endemic human coronaviruses (HCoVs) accounted for up to 15% of all viral acute respiratory infections [6–11].

CoVs are enveloped, positive-sense RNA viruses [12]. The genomes of HCoVs range from approximately 27.3 kb (HCoV-229E) to 30.7 kb (HCoV-OC43) in length [4, 13, 14] and encode four major structural proteins: nucleocapsid (N), membrane (M), envelope (E), and spike (S) [12]. Among these, the spike protein plays a central role in viral entry into host cells [15]. The non-structural proteins of HCoVs are encoded by ORFs 1a and 1b, which together account for approximately two-thirds of the genome and are essential for proteolytic processing, genome replication, and subgenomic mRNA synthesis [12].

HCoVs usually cause mild upper respiratory tract infections characterized by symptoms such as rhinitis, cough, headache and fever [8, 16–20]. However, lower respiratory tract infections and severe clinical courses have also been reported in literature [16, 21–25]. HCoVs exhibit pronounced winter seasonality in temperate regions of both the Northern and Southern Hemispheres, whereas such seasonality is substantially attenuated or absent in tropical regions [26].

Data on viral circulation and clinical manifestations of HCoV infections in Germany are limited. In particular, longitudinal studies covering all HCoVs are lacking. This study therefore aimed to characterize the epidemiology and clinical significance of HCoV-infections in adult patients over 11 seasons (2012 to 2021/2022) at a tertiary care hospital in Germany.

## Materials and methods

### Sample collection

Between January 2012 and September 2022, a total of 29,104 respiratory samples from 15,983 adult (≥ 18 years) inpatients and outpatients were routinely submitted and tested for viral respiratory infections at University of Leipzig Medical Center, Germany.

The analyzed specimens comprised nasal and/or naso-or opharyngeal swabs (26.7%, *n* = 7,776), throat rinsing fluids (52.6%, *n* = 15.300), bronchoalveolar lavage fluids (14.6%, *n* = 4,262), tracheal secretions (5.5%, *n* = 1,587), and sputum samples (0.6%, *n* = 187). Testing was initiated at the discretion of the responsible physician. To avoid bias caused by follow-up samples, patients retested within six weeks after initial detection were considered as a single case. A respiratory season was defined as starting on October 1st and ending on September 30th of the following year.

### Study population and clinical data

For this retrospective observational study, data relating to underlying medical conditions, comorbidities, and clinical parameters from the timepoint of HCoV detection were retrieved from patient charts. Where clinical information was missing, the notation “(n/total)” denotes the number of cases fulfilling the respective criterion (n) relative to the number of cases with available data for that parameter (total).

Body temperature > 38.0 °C was categorized as fever. The classification of URTIs and LRTIs was based on the International Statistical Classification of Diseases and Related Health Problems (ICD-11-WHO) [27] as well as the diagnoses and information listed in the patients’ records. Patients with any of the following conditions were considered immunocompromised: receiving active chemotherapy for neoplastic disease, chronic neutropenia (< 1,500/µL for more than three months), receiving steroids or other immunomodulatory medications prior to visit/admission, aplasia of the thymus gland, or a reported HIV infection classified as CDC Stage 3 (CD4 < 200 cells/µL or CD4 < 14% or AIDS-defining illness) [28]. Furthermore, comorbidities were classified according to the age-adjusted Charlson Comorbidity Index (CCI) [29]. Asthma, COPD, and Asthma-COPD Overlap Syndrome (ACOS) were classified together as obstructive lung disease (OLD) [30]. Anticholinergics and β2-adrenergic agonists (i.e. ipratropium bromide and salbutamol) were classified as bronchodilators.

An infection was considered nosocomial if the patient had no respiratory symptoms on admission and developed first symptoms of an acute respiratory infection (fever > 38 °C or self-reported, newly developed cough or dyspnea) at least 72 h after admission, or was readmitted with symptoms of an acute respiratory infection within 48 h after discharge.

Bacterial and fungal pathogens were cultured using standard microbiological techniques and were classified as co-infections if detected in respiratory samples or blood. Viral co-infections were assessed by multiplex testing for respiratory viruses (see below) and by established routine laboratory protocols for CMV [31]. SARS-CoV-2 was detected using a commercially available RT-PCR assay on the Alinity m system (Abbott, Chicago, IL, USA) targeting the viral RdRp- and N-genes.

### Nucleic acid extraction and HCoV detection

Total nucleic acid (NA) was extracted from 200 µl of respiratory samples using the DNA and Viral NA Small Volume Kit on the MagNA Pure 96 instrument (both Roche, Mannheim, Germany) according to the manufacturer’s instructions. Extracted NAs were aliquoted and stored at -80 °C until further use. The presence of genomes of common respiratory viruses, including influenza A and B viruses, RSV-A and RSV-B, parainfluenza viruses 1–4, HCoV-NL63, HCoV-229E, HCoV-OC43, HCoV-HKU1, human metapneumoviruses, adenoviruses, human bocaviruses, rhinoviruses, and enteroviruses was assessed using a multiplex panel assay (NxTAG^®^ Respiratory Pathogen Panel, Luminex Molecular Diagnostics, Inc., a DiaSorin Company, Toronto, ON, Canada) according to the manufacturer’s instructions. Samples positive for at least one of the four HCoV targets were subjected to further analysis (case).

### Statistical analysis

Statistical analyses were performed using IBM SPSS Statistics for Windows, version 29.0 (IBM Corp., Armonk, NY, USA). Continuous variables were expressed as means or medians (range), and categorical data as frequencies (percentages). A Mann–Whitney U test was used to compare continuous variables. Categorial variables were compared using the chi-square test or Fisher’s exact test, as appropriate. All tests were two-tailed, and a p-value of < 0.05 was considered statistically significant. For post-hoc pairwise comparisons of column proportions, a Bonferroni correction for multiple comparisons was applied.

## Results

### Seasonality

In total, HCoV was detected by RT-PCR in 1,048 samples representing 469 unique cases. HCoVs detections exhibited a recurrent seasonal pattern from 2012 to 2020, with the majority of cases occurring during the winter and spring months and only sporadic detections during summer. Typically, detections began in late autumn (November–December), peaked between January and March, and declined steadily during spring. By summer (July–September), detections were rare or absent.

In most seasons, the betacoronaviruses HCoV-HKU1 and HCoV-OC43 peaked between December and February, whereas the alphacoronaviruses HCoV-NL63 and HCoV-229E tended to peak around March and April.

**Fig. 1:**
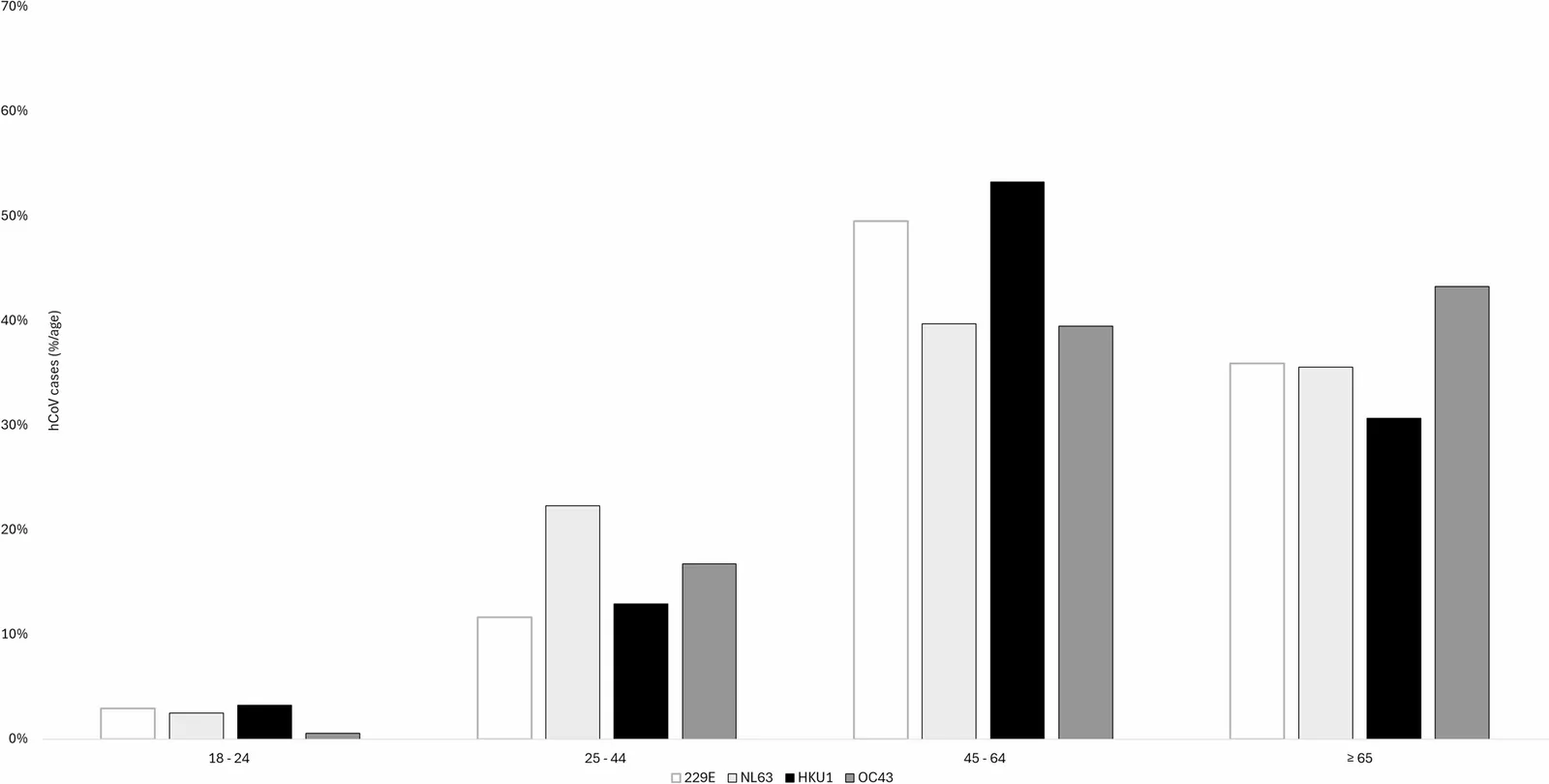
Relative distribution of HCoV cases (*n* = 469) by age. The bars represent the relative number of cases for each species detected in the indicated age group

While alphacoronavirus HCoV-NL63 and betacoronavirus HCoV-OC43 were detected in every season, HCoV-229E was absent in 2013/2014 and 2017/2018, and HCoV-HKU1 in 2016/2017. Overall, HCoV-OC43 was the most frequently detected species, accounting for 38.6% (181/469) of all cases, followed by HCoV-NL63 with 25.6% (120/469), HCoV-229E with 22.4% (105/469), and HCoV-HKU1 with 13.4% (63/469). The relative numbers of adult HCoV cases detected between 2012 and 2022, stratified by species and age-group are shown in Fig. 1.

**Fig. 2:**
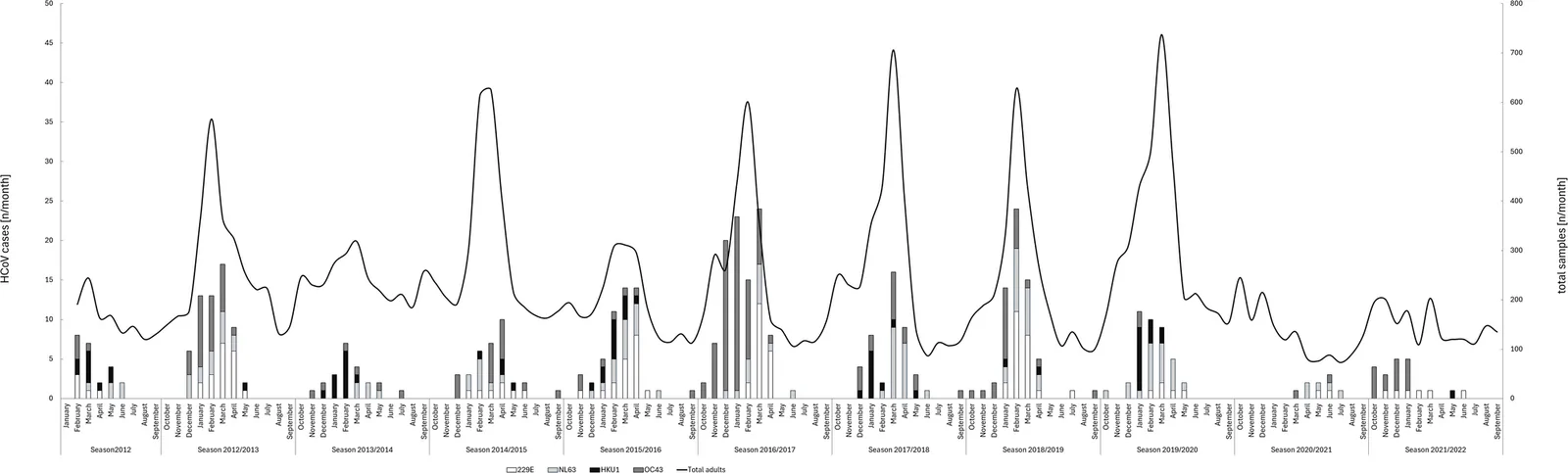
Monthly distribution of newly detected HCoV cases (*n* = 469), stratified by species (HCoV-HKU1, HCoV-229E, HCoV-NL63, and HCoV-OC43), and the total number of respiratory samples tested. Bars represent the absolute number of newly detected HCoV cases per month by species (left y-axis), whereas the dashed line represents the total number of respiratory samples tested per month (right y-axis). The x-axis is displayed according to the predefined respiratory seasons, each spanning from October 1 to September 30

Detections of HCoV-OC43 and HCoV-HKU1 peaked in alternating winter seasons, with each virus predominating approximately every other season. HCoV-HKU1 was predominant in 2012 and seasons 2013/2014, 2015/2016, 2017/2018, 2019/2020, whereas HCoV-OC43 predominated in 2012/2013, 2014/2015, 2016/2017, and 2018/2019 (Fig. 2, species-specific data are provided in Supplementary Figures S1a -S1d and Supplementary Table S1).

The two alphacoronaviruses, HCoV-NL63 and HCoV-229E, also showed a biennial detection pattern, although less pronounced than that observed for the betacoronaviruses. HCoV-NL63 was detected every season, whereas HCoV-229E was absent in 2013/2014 and 2017/2018, each following a season with high HCoV-229E activity.

From the 2020/2021 season onward, the detection patterns of HCoVs changed. Between February 2021 and July 2022, all HCoVs were detected at low frequency, with HCoV-OC43 being the predominant species.

### Study population and clinical features

The study population included adult patients aged 18 years and older. Across all HCoV cases, the median age was 60 years (IQR, 50–70 years). Neither median age nor sex distribution differed significantly between the four HCoV species. Patient characteristics and clinical parameters associated with HCoV infection are presented in Table 1. The age-adjusted CCI did not differ significantly between the four groups.

**Table 1:** Study population characteristics and clinical features of adult patients with HCoV infection

		229E 22.4% (105/469)	NL63 25.6% (120/469)	HKU1 13.4% (63/469)	OC43 38.6% (181/469)	Total 469	*p*-Value
**Study population**							
Female	[% (n/total)]	40 (42/105)	45 (54/120)	25.4 (16/63)	38.7 (70/181)	38.8 (182/469)	(n.s.)
Male	[% (n/total)]	60 (63/105)	55 (66/120)	74.6 (47/63)	61.3 (111/181)	61.2 (287/469)	
Age [years]	[median (IQR)]	60 (53–70)	58 (44.5–68)	59 (51–65)	61 (50–74)	60 (50–70)	n.s
Outpatients	[% (n/total)]	32 (33/103)	37.8 (45/119)	20 (12/60)	29.8 (54/181)	31.1 (144/463)	(n.s.)
Inpatients	[% (n/total)]	68 (70/103)	62.2 (74/119)	80 (48/60)	70.2 (127/181)	68.9 (319/463)	
Length of stay [days]	[median (IQR)]	12.5 (5.75-22)	15.5 (7–28)	17 (8–27)	13 (7–22)	14 (6–24)	n.s.
Hospital-acquired Infection	[% (n/total)]	22.3 (23/103)	15.1 (18/119)	35 (21/60)	21 (38/181)	21.6 (100/463)	**0.025**
**Co-morbidities and risk factors**							
OLD	[% (n/total)]	23.3 (24/103)	18.4 (21/114)	20.3 (12/59)	29.6 (53/179)	24.2 (110/455)	n.s.
Asthma	[% (n/total)]	4.9 (5/103)	2.6 (3/114)	1.7 (1/59)	5 (9/179)	4 (18/455)	n.s.
COPD	[% (n/total)]	18.4 (19/103)	15.8 (18/114)	20.3 (12/59)	25.7 (46/179)	20.9 (95/455)	n.s.
Structural lung disease	[% (n/total)]	8.7 (9/103)	11.4 (13/114)	5.1 (3/59)	11.2 (20/179)	9.9 (45/455)	n.s.
Lung transplant	[% (n/total)]	17.3 (18/104)	12.3 (14/114)	11.7 (7/60)	5.6 (10/179)	10.7 (49/457)	**0.019**
Pulmonary hypertension	[% (n/total)]	3.9 (4/103)	6.1 (7/114)	3.4 (2/59)	6.1 (11/179)	5.3 (24/455)	n.s.
Congestive heart failure	[% (n/total)]	5.8 (6/103)	6.1 (7/114)	8.5 (5/59)	7.8 (14/179)	7 (32/455)	n.s.
Arterial hypertension	[% (n/total)]	51.5 (53/103)	50 (57/114)	45.8 (27/59)	57 (102/179)	52.5 (239/455)	n.s.
Coronary heart disease	[% (n/total)]	13.6 (14/103)	14.9 (17/114)	10.2 (6/59)	13.4 (24/179)	13.4 (61/455)	n.s.
Diabetes	[% (n/total)]	22.3 (23/103)	15.8 (18/114)	23.7 (14/59)	15.1 (27/179)	18 (82/455)	n.s.
Chronic renal failure	[% (n/total)]	24.3 (25/103)	20.2 (23/114)	22 (13/59)	22.3 (40/179)	22.2 (101/455)	n.s.
At least 1 Malignancy < 5 years	[% (n/total)]	51.5 (53/103)	55.3 (63/114)	64.4 (38/59)	47.5 (85/179)	52.5 (239/455)	n.s.
Leukemia	[% (n/total)]	16.5 (17/103)	22.8 (26/114)	18.6 (11/59)	26.3 (47/179)	22.2 (101/455)	n.s.
Lymphoma	[% (n/total)]	21.4 (22/103)	24.6 (28/114)	35.6 (21/59)	13.4 (24/179)	20.9 (95/455)	**0.002**
Solid tumor	[% (n/total)]	9.7 (10/103)	7.9 (9/114)	15.3 (9/59)	5.6 (10/179)	8.4 (38/455)	n.s.
Metastatic tumor	[% (n/total)]	4.9 (5/103)	4.4 (5/114)	1.7 (1/59)	3.9 (7/179)	4 (18/455)	n.s.
Immunosuppression	[% (n/total)]	46.6 (48/103)	51.8 (59/114)	50 (30/60)	47.5 (85/179)	48.7 (222/456)	n.s.
Stem cell transplantation*	[% (n/total)]	19.4 (20/103)	31.6 (36/114)	33.9 (20/59)	25.1 (45/179)	26.6 (121/455)	n.s.
Neurological diseases	[% (n/total)]	7.8 (8/103)	8.8 (10/114)	3.4 (2/59)	8.9 (16/179)	7.9 (36/455)	n.s.
Charlson-score	[median (IQR)]	4 (3–6)	4 (2–6)	4 (2–6)	4 (2–6)	4 (2–6)	n.s.
**Co-infections**							
Any Co-infection	[% (n/total)]	27.2 (28/103)	22.4 (26/116)	28.8 (17/59)	26.7 (48/180)	26 (119/458)	n.s.
Viral only	[% (n/total)]	15.5 (16/103)	10.3 (13/116)	6.8 (4/59)	10 (18/180)	10.9 (50/458)	n.s.
Bacterial only	[% (n/total)]	7.8 (8/103)	7.8 (9/116)	13.6 (8/59)	12.8 (23/180)	10.5 (48/458)	n.s.
Fungal only	[% (n/total)]	1.9 (2/103)	1.7 (2/116)	3.4 (2/59)	1.1 (2/180)	1.7 (8/458)	n.s.
Combined	[% (n/total)]	1.9 (2/103)	2.6 (3/116)	6.8 (4/59)	3.3 (6/180)	3.3 (14/458)	n.s.
**Clinical presentation**							
Fever	[% (n/total)]	35.2 (25/71)	45.6 (36/79)	26.1 (12/46)	40.9 (52/127)	38.7 (125/323)	n.s.
Dyspnea	[% (n/total)]	34.3 (24/70)	30.3 (27/89)	22.4 (11/49)	48.1 (63/131)	36.9 (125/339)	**0.004**
URTI	[% (n/total)]	49.2 (30/61)	61.4 (54/88)	44.7 (21/47)	53.4 (62/116)	53.5 (167/312)	n.s.
LRTI	[% (n/total)]	31.9 (30/94)	28 (30/107)	33.3 (19/57)	45.3 (77/170)	36.4 (156/428)	**0.018**
Pneumonia	[% (n/total)]	22.3 (21/94)	22.4 (24/107)	26.3 (15/57)	27.1 (46/170)	24.8 (106/428)	n.s.
Bronchitis	[% (n/total)]	6.4 (6/94)	4.7 (5/107)	7 (4/57)	8.8 (15/170)	7 (30/428)	n.s.
Exacerbation of OLD	[% (n/total)]	8,5 (8/94)	4.7 (5/107)	5.3 (3/57)	17.6 (30/170)	10.7 (46/428)	**0.002**
ARDS	[% (n/total)]	3.9 (4/103)	5.2 (6/115)	5.1 (3/59)	1.7 (3/179)	3.5 (16/456)	n.s.
ICU stay	[% (n/total)]	18.4 (19/103)	17.6 (21/119)	30.5 (18/59)	25.4 (46/181)	22.5 (104/462)	n.s.
Length of ICU-stay [days]	[median (IQR)]	3 (2–7)	7 (4–14)	8.5 (2-12.5)	5 (2-8.25)	5 (2–10)	n.s
Ventilation	[% (n/total)]	11.7 (12/103)	14.3 (17/119)	16.9 (10/59)	18.2 (33/181)	15.6 (72/462)	n.s.
Invasive	[% (n/total)]	10.7 (11/103)	10.9 (13/119)	15.3 (9/59)	9.9 (18/181)	11 (51/462)	n.s.
Non-invasive	[% (n/total)]	1 (1/103)	3.4 (4/119)	1.7 (1/59)	8.3 (15/181)	4.5 (21/462)	n.s.
Bronchodilatators	[% (n/total)]	14.3 (14/98)	12.4 (14/113)	8.5 (5/59)	13.1 (23/175)	12.6 (56/445)	n.s.
Systemic Predninsolone administration	[% (n/total)]	4.1 (4/98)	9.7 (11/113)	5.1 (3/59)	16 (28/175)	10.3 (46/445)	**0.007**
In-hospital mortality	[% (n/total)]	9.7 (10/103)	7.6 (9/119)	18.6 (11/59)	6.6 (12/181)	9.1 (42/469)	**0.042**

Data are presented as frequencies (% [n/total]) or as median (IQR). In the presence of missing data, “(n/total)” indicates the number of cases meeting the respective criterion (n) out of the number of cases with available data for that parameter (total). p-values refer to overall comparisons across the four HCoV species. Significant post-hoc pairwise comparisons are reported in the corresponding Results section. Continuous variables were compared using the Mann–Whitney U test, and categorical variables using the chi-square test or Fisher’s exact test, as appropriate. Abbreviations: CCI, Charlson comorbidity index; HFNC, high-flow nasal cannula; ICU, intensive care unit; IQR, interquartile range; LRTI, lower respiratory tract infection; n.s., not significant; OLD, obstructive lung disease; syst., systemic; URTI, upper respiratory tract infection. *including allogeneic and autologous transplantation and one case of CAR-T cell therapy

HCoV-HKU1 was more frequently detected in patients with lymphoma (35.6%, *p* = 0.002) and was associated with higher in-hospital mortality (18.6%, *p* = 0.042) and a higher proportion of hospital-acquired infections (35.0%, *p* = 0.025). HCoV-229E infections were more common in patients with prior lung transplantation (17.3%, *p* = 0.019).

HCoV-OC43 infection was more frequently associated with dyspnea (48.1%, *p* = 0.004), lower respiratory tract infection (45.3%, *p* = 0.018), exacerbation of obstructive lung disease (OLD; 17.6%, *p* = 0.002), and administration of systemic prednisolone (16%, *p* = 0.007) compared with the other HCoV species. In contrast, a significantly smaller proportion of HCoV-OC43 infections were observed in lung-transplant patients (5.6%, *p* = 0.019) and in patients with lymphoma (13,4%, *p* = 0.002).

### Co-infections

In total, 119 co-infections (26%) were identified, including 51 viral (10.9%) 48 bacterial (10.5%), and 8 fungal (1.7%) pathogens (Supplementary Table S2). In 14 cases (3.1%) co-infections with more than one pathogen were observed. The most common bacterial pathogens were *Staphylococcus aureus* (*n* = 18), *Haemophilus influenzae* (*n* = 8), and *Streptococcus pneumoniae* (*n* = 8). Among viral co-pathogens, RSV (*n* = 12), influenza A/H1N1 (*n* = 10), influenza A/H3N2 (*n* = 10), and rhinovirus (*n* = 10) were most frequently detected. Among fungal infections *Aspergillus spp.* (*n* = 3) and *Pneumocystis jiroveci* (*n* = 3) predominated. No significant difference was observed in the frequency of co-infections among the four HCoV species (Supplementary Tables S3 and S4). However, LRTI (*p* < 0.001) occurred more frequently in patients with at least one co-infection than in those with HCoV infection alone (Supplementary Table S5). In addition, co-infections were more frequently associated with markers of higher disease severity, i.e. dyspnea (*p* < 0.001), need for ventilation (*p* = 0.001), and an ICU stay (*p* < 0.001). The frequency of bacterial and mixed infections was higher in patients with LRTI (Supplementary Table S6).

## Discussion

Human endemic coronaviruses are among the most common pathogens causing respiratory tract infections and are distributed worldwide [26, 32, 33]. In temperate regions of both hemispheres, they exhibit a marked winter seasonality. In the Northern Hemisphere, and in line with our findings, detections of the two betacoronaviruses HCoV-HKU1 and HCoV-OC43 have previously been reported to peak in December and January, whereas the alphacoronaviruses HCoV-229E and HCoV-NL63 tend to peak between February and April [16, 26, 34]. In contrast, in the Southern Hemisphere, peaks occur between May and September, while tropical regions show weaker or less clearly defined seasonal patterns [26, 35]. The frequency of HCoV detection varies slightly depending on the study location [35]. In a surveillance study from Central Europe conducted between 2010 and 2019, HCoV-OC43 was detected most frequently, followed by HCoV-NL63, HCoV-229E, and HCoV-HKU1 [36], which is consistent with our findings. However, regional differences may be observed, even within the same country. In the present study, HCoV-HKU1 was detected between 2012 and 2015 and was the predominant betacoronavirus in the 2012 and 2013/2014 seasons. In contrast, HCoV-HKU1 was not detected in another Central European study conducted between 2010 and 2015 [36].

Studies covering several consecutive seasons have demonstrated alternating circulation patterns for the alphacoronaviruses HCoV-NL63 and HCoV-229E, as well as for the betacoronaviruses HCoV-OC43 and HCoV-HKU1 [16, 37]. During our study period, this biennal circulation pattern was particularly pronounced for HCoV-OC43 and HCoV-HKU1, whereas it was less evident for HCoV-NL63 and HCoV-229E. The underlying reasons for this distinct circulation pattern are not yet fully understood but may, at least in part, be related to cross-immunity between species [38]. In addition, studies on the duration of immunity following HCoV infection suggest that protective immunity persists only for a limited period [39–41]. Waning population immunity over time may therefore facilitate the emergence of new epidemic waves [42–44].

During the SARS-CoV-2 pandemic, a broad range of non-pharmaceutical interventions (NPIs) was introduced in Germany in March 2020 [45]. These included several nationwide lockdowns between March and April 2020 and between December 2020 and March 2021, as well as the closure of schools and daycare centers [46]. In addition to these legal restrictions, the pandemic profoundly affected societal behavior, including mask-wearing in public and increased working from home [47]. NPIs began to be gradually relaxed in May 2021 and were linked to the incidence of SARS-CoV-2 infections. With the onset of the SARS-CoV-2 pandemic in the 2020/2021 season and the implementation of containment measures in Germany, the previously observed epidemiological pattern of HCoVs disappeared. Notably, the first HCoV cases in the 2020/2021 season were not detected until March 2021, and the typical January/February peak of the betacoronaviruses was absent. Since then, HCoVs have been detected almost continuously, including during the summer months. However, the distribution of the four HCoV species remained broadly similar. In particular, HCoV-OC43 continued to be the predominant species, whereas HCoV-HKU1 was not detected again until May 2022. A disruption of seasonal circulation in temperate climates was also observed for other viral infections, such as RSV [48, 49] and influenza [50], whereas viruses with a year-round circulation, such as rhinoviruses/enterovirus and adenoviruses, were less affected [51]. Further studies are needed to assess the possible impact of SARS-CoV-2-mediated cross-immunity on HCoV circulation [52–54].

Depending on the patient cohort, endemic coronaviruses in humans may be associated with a broad spectrum of clinical manifestations, ranging from common colds and flu-like illnesses [9, 10, 16, 17, 55, 56] to asymptomatic or subclinical infections [57–59]. However, species-specific differences in clinical presentations have also been reported. For example, HCoV-OC43 has been detected in the central nervous system, including in brain biopsies specimens, in pediatric patients with fatal encephalitis and ADEM [60, 61]. HCoV-NL63 has been more frequently associated with croup in pediatric patients [62], whereas HCoV-HKU1 in pediatric patients has been linked to febrile seizures [18]. Furthermore, the co-infection status needs to considered for the assessment of the clinical presentation (see below).

In our cohort, exacerbation of OLD was associated with HCoV-OC43 infection. As exacerbations of OLD are typically accompanied by acute dyspnea, this may explain the higher frequency dyspnea observed in patients with HCoV-OC43 infection. In the same context, the increased use of systemic prednisolone in patients with HCoV-OC43 infection is consistent with the higher rate of OLD exacerbations. The overall number of LRTI cases was also higher in HCoV-OC43 than in infections with the other HCoV species, most likely reflecting the increased frequency of OLD exacerbations. Despite the significant differences in LRTI and OLD exacerbations associated with HCoV-OC43, no longer hospital stays or higher in-house mortality were observed compared with the other species. It is possible that the higher proportion of patients with malignancies in the HCoV-HKU1 group masked differences relative to HCoV-OC43 in the predominantly hospitalized cohort. While HCoV-OC43-associated OLD exacerbations were not linked to prolonged hospitalization or increased mortality, both outcomes were more frequently observed in HCoV-HKU1 infection, likely reflecting the greater vulnerability of patients with malignant disease.

HCoVs have generally been implicated, along with other circulating respiratory pathogens, in the development of OLD exacerbation [63, 64]. However, compared with influenza, HCoV infections were not associated with a significant deterioration in FEV1. The need for hospitalizations was similar in patients with influenza and those with HCoV infection [65].

HCoV-HKU1 was more frequently detected in patients with lymphoma (35.6%, *p* = 0.002) and was associated with higher in-hospital mortality (18.6%, *p* = 0.042) and a higher proportion of hospital-acquired infections (35.0%, *p* = 0.025). Compared with HCoV-OC43, for which the mortality was lower (6.6%, *p* = 0.042), these findings may indicate a less favorable clinical course in patients with HCoV-HKU1 infection. Similar, although non-significant, trends were observed in patients with solid tumors and in stem cell transplant recipients. In contrast, HCoV-229E infections were significantly more common in patients with prior lung transplantation and subsequent active immunosuppression (17.3%, *p* = 0.019), whereas HCoV-OC43 was less prevalent in this subgroup. The reasons for these differences remain unclear and may reflect differences in the degree or type of immunosuppression, among other factors.

Previous studies have reported heterogeneous findings regarding associations between specific HCoV species, comorbidities and clinical outcomes. Several case reports suggest that HCoV-229E and HCoV-HKU1 may cause more severe disease in vulnerable patients [21, 66–70]. One systematic study found that HCoV-229E infections were more frequently observed in immunosuppressed patients [16] and could even progress to LTRI [71]. In addition, several studies have reported associations between seasonal HCoV infections and a more severe disease course or disease progression, including correlations with chronic lung allograft dysfunction [72, 73] and an increased risk of progression to lower respiratory tract infection (LRTI) in patients receiving systemic prednisolone [74]. However, clear evidence for species-specific differences in clinical severity remains lacking [16, 75].

Across published cohorts, co-infections with at least one additional respiratory pathogen have been reported in 12%–34.6% of patients [34, 37, 76, 77]. In adults, HCoV co-infections have predominantly been documented as viral, with rhinovirus and RSV being the most common co-pathogens [16, 34], whereas bacterial co-infections remain insufficiently studied in this population.

In our study, 26.0% of coronaviruses were associated with at least one additional pathogen. Moreover, LRTIs occurred more frequently in patients with at least one respiratory co-infection (*p* < 0.001). Patients with co-infections were also more likely to require invasive or non-invasive ventilation (*p* < 0.001).

As demonstrated for other respiratory viruses, bacterial co-infections may substantially contribute to clinical severity, although distinguishing bacterial colonization from true infection remains challenging. Across multiple cohorts, viral respiratory infections have most commonly been associated with *Streptococcus pneumoniae*,* Haemophilus influenzae*,* Pseudomonas aeruginosa*,* Moraxella catarrhalis*,* Mycoplasma pneumoniae*, and *Staphylococcus aureus* [78–80]. For other respiratory viruses, including RSV, parainfluenza viruses, influenza viruses, and hMPV, multiple pathomechanisms have been described in cell culture and animal models [81]. These include enhanced bacterial adherence to the airway epithelium (e.g., *H. influenzae*, *S. pneumoniae*) [82–84], disruption of innate and adaptive mucosal immunity through impaired phagocyte function, altered cytokine responses [85, 86], and reduced antimicrobial peptide expression; as well as host priming for bacterial pneumonia through exaggerated inflammation, alveolar damage, and impaired microbial clearance, thereby promoting secondary superinfection [78]. For HCoV-NL63, one in vitro study suggested that HCoV-NL63 infection can increase *S. pneumoniae* adherence to infected airway epithelial cells, including primary differentiated human airway epithelium, indicating a potential mechanism for post-viral bacterial superinfection [79]. Additional observations suggest that endemic human coronaviruses may dysregulate interferon-mediated immunity in the respiratory tract and thereby contribute to conditions that favor bacterial superinfection [80, 81]. In our cohort, *S. aureus* was the most frequently detected bacterial co-pathogen. Whether this reflects true co-infection or synergistic interactions between *S. aureus* and HCoV remains unclear and warrants further investigation. Characteristics of the patient cohort, such as ICU treatment or mechanical ventilation, may also play an important role. In line with this, co-infections were observed significantly more frequently among ICU patients (*p* < 0.001).

As reported in a previous study conducted at our institution, the mean age of patients with HCoV was comparable to that of patients with rhinovirus (RV) infection (54.8 years) and RSV [48, 87]. Lower respiratory tract infections were documented in 41.3% (159/385) of HCoV cases and thus occurred at a frequency intermediate between RV (28.6%, 70/254) and RSV (50.8%, 90/177) infections. ICU admission was required in 8.8% (27/284) of RV cases, 25.0% (49/196) of RSV cases, and 20.2% (80/396) of HCoV cases. Mechanical ventilation was required in 10.6% (30/284) of RV infections, 8.1% (16/196) of RSV infections, and 15.6% (72/462) of HCoV infections. Co-infections were observed in 21.1% (60/284) of RV cases and 20.9% (41/196) of RSV cases, compared with 29.2% (134/459) in the present HCoV cohort.

Several limitations of this study should be acknowledged. First, a selection toward more severe cases is likely, as sampling was performed at a tertiary care hospital, where patients tend to be more severely ill, as reflected by the high numbers of pneumonia cases and ICU admissions. Consequently, the high rate of LRTI should be interpreted with caution, since the selective testing approach likely led to underdetection of mild or asymptomatic infections. Second, due to the study design, only associations could be identified and no causal inferences can be made.

## Conclusions

This study characterizes the epidemiology and clinical spectrum of HCoV infections in adult patients treated at a tertiary care university hospital in Germany. The observed circulation patterns align broadly consistent with previous reports, highlighting the complexity of HCoVs epidemiology. However, further research is needed to clarify the factors underlying the observed epidemiological patterns. In addition, more studies are required to identify determinants of severe disease progression, particularly in vulnerable and immunocompromised patient, and the role of co-infections, especially bacterial co-infections. Given the limited size of our cohort and its predominance of hospitalized patients, future studies should include larger and outpatient populations to allow a more comprehensive assessment of the overall disease burden. Finally, it remains essential to evaluate how the COVID-19 pandemic has influenced the epidemiology and clinical relevance of endemic HCoVs.

## Supplementary Information

Below is the link to the electronic supplementary material.

Supplementary Material 1: Table S1: Absolute species-specific coronavirus detections stratified by season and month, Figure S1a: Monthly distribution of new HCoV-229E cases; Figure S1b: Monthly distribution of new HCoV-NL63 cases, Figure S1c: Monthly distribution of new HCoV-HKU1 cases; Figure S1d: Monthly distribution of new HCoV-OC43 cases, Table S2: Co-infecting pathogens, Table S3: Stratification of co-infecting viral pathogens, Table S4: Stratification of co-infecting bacterial pathogens, Table S5: Clinical characteristics and outcomes in patients with co-infection, Table S6: Distribution of co-infection types according to LRTI status

## Data Availability

The data that support the findings of this study are not openly available due to reasons of participant confidentiality and are available from the corresponding author upon reasonable request. Data are located in controlled access data storage at Leipzig University.
