# Supplementary material for: A decade of endemic human coronaviruses: epidemiological patterns and clinical significance in an adult cohort at a tertiary care hospital in Germany
Source: BMC Infect Dis. 2026 Sep 18;26:1706. doi: 10.1186/s12879-026-14377-1 (PMC13599198; doi:10.1186/s12879-026-14377-1)
Supplement: Supplementary file 1 [file 12879_2026_14377_MOESM1_ESM.docx]

**A Decade of Endemic Human Coronaviruses: Epidemiological Patterns and Clinical Significance in an Adult Cohort at a Tertiary Care Hospital in Germany**

Christian Propach, Mario Hönemann, Vladan Vučinić, Armin Frille, Tom Berthold, Sandra Bergs, Melanie Maier and Corinna Pietsch

**Supplementary Material**

**Table S1.** Absolute species-specific coronavirus detections stratified by season and month.

| **Seasons** | **months** | **229E** | **NL63** | **HKU1** | **OC43** | **Total** | **Total per season** |
| --- | --- | --- | --- | --- | --- | --- | --- |
|  | January | 0 | 0 | 0 | 0 | 0 | 15 |
|  | February | 3 | 0 | 2 | 3 | 8 |  |
|  | March | 1 | 1 | 4 | 1 | 7 |  |
|  | April | 1 | 0 | 1 | 0 | 2 |  |
| **Season 2012** | May | 0 | 2 | 2 | 0 | 4 |  |
|  | June | 0 | 2 | 0 | 0 | 2 |  |
|  | July | 0 | 0 | 0 | 0 | 0 |  |
|  | August | 0 | 0 | 0 | 0 | 0 |  |
|  | September | 0 | 0 | 0 | 0 | 0 |  |
|  | October | 0 | 0 | 0 | 0 | 0 | 41 |
|  | November | 0 | 0 | 0 | 0 | 0 |  |
|  | December | 0 | 3 | 0 | 3 | 6 |  |
|  | January | 2 | 2 | 0 | 9 | 13 |  |
|  | February | 3 | 3 | 0 | 7 | 13 |  |
| **Season 2012/2013** | March | 7 | 4 | 0 | 6 | 17 |  |
|  | April | 6 | 2 | 0 | 1 | 9 |  |
|  | May | 1 | 0 | 1 | 0 | 2 |  |
|  | June | 0 | 0 | 0 | 0 | 0 |  |
|  | July | 0 | 0 | 0 | 0 | 0 |  |
|  | August | 0 | 0 | 0 | 0 | 0 |  |
|  | September | 0 | 0 | 0 | 0 | 0 |  |
|  | October | 0 | 0 | 0 | 0 | 0 | 32 |
|  | November | 0 | 0 | 0 | 1 | 1 |  |
|  | December | 0 | 0 | 1 | 1 | 2 |  |
|  | January | 0 | 0 | 3 | 0 | 3 |  |
|  | February | 0 | 0 | 6 | 1 | 7 |  |
| **Season 2013/2014** | March | 0 | 2 | 1 | 1 | 4 |  |
|  | April | 0 | 2 | 0 | 0 | 2 |  |
|  | May | 0 | 1 | 0 | 1 | 2 |  |
|  | June | 0 | 0 | 0 | 0 | 0 |  |
|  | July | 0 | 0 | 0 | 1 | 1 |  |
|  | August | 0 | 0 | 0 | 0 | 0 |  |
|  | September | 0 | 0 | 0 | 0 | 0 |  |

|  | October | 0 | 0 | 0 | 0 | 0 | 35 |
| --- | --- | --- | --- | --- | --- | --- | --- |
|  | November | 0 | 0 | 0 | 0 | 0 |  |
|  | December | 0 | 0 | 0 | 3 | 3 |  |
|  | January | 1 | 2 | 0 | 0 | 3 |  |
|  | February | 1 | 4 | 1 | 0 | 6 |  |
| **Season 2014/2015** | March | 1 | 1 | 0 | 5 | 7 |  |
|  | April | 2 | 1 | 2 | 5 | 10 |  |
|  | May | 1 | 0 | 1 | 0 | 2 |  |
|  | June | 1 | 0 | 0 | 1 | 2 |  |
|  | July | 0 | 0 | 0 | 0 | 0 |  |
|  | August | 0 | 0 | 0 | 0 | 0 |  |
|  | September | 0 | 0 | 0 | 1 | 1 |  |
|  | October | 0 | 0 | 0 | 0 | 0 | 38 |
|  | November | 1 | 0 | 0 | 2 | 3 |  |
|  | December | 0 | 1 | 1 | 0 | 2 |  |
|  | January | 1 | 1 | 2 | 1 | 5 |  |
|  | February | 2 | 3 | 5 | 1 | 11 |  |
| **Season 2015/2016** | March | 5 | 5 | 3 | 1 | 14 |  |
|  | April | 8 | 4 | 1 | 1 | 14 |  |
|  | May | 1 | 0 | 0 | 0 | 1 |  |
|  | June | 0 | 1 | 0 | 0 | 1 |  |
|  | July | 0 | 0 | 0 | 0 | 0 |  |
|  | August | 0 | 0 | 0 | 0 | 0 |  |
|  | September | 0 | 0 | 0 | 1 | 1 |  |
|  | October | 0 | 0 | 0 | 2 | 2 | 46 |
|  | November | 0 | 0 | 0 | 7 | 7 |  |
|  | December | 0 | 1 | 0 | 19 | 20 |  |
|  | January | 0 | 1 | 0 | 22 | 23 |  |
|  | February | 2 | 3 | 0 | 10 | 15 |  |
| **Season 2016/2017** | March | 12 | 5 | 0 | 7 | 24 |  |
|  | April | 6 | 1 | 0 | 1 | 8 |  |
|  | May | 0 | 0 | 0 | 0 | 0 |  |
|  | June | 0 | 1 | 0 | 0 | 1 |  |
|  | July | 0 | 0 | 0 | 0 | 0 |  |
|  | August | 0 | 0 | 0 | 0 | 0 |  |
|  | September | 0 | 0 | 0 | 0 | 0 |  |
|  | October | 0 | 0 | 0 | 0 | 0 | 53 |
|  | November | 0 | 0 | 0 | 0 | 0 |  |
|  | December | 0 | 0 | 1 | 3 | 4 |  |
|  | January | 0 | 0 | 6 | 2 | 8 |  |
|  | February | 0 | 1 | 1 | 0 | 2 |  |
| **Season 2017/2018** | March | 0 | 9 | 1 | 6 | 16 |  |
|  | April | 0 | 7 | 0 | 2 | 9 |  |
|  | May | 0 | 0 | 1 | 2 | 3 |  |
|  | June | 0 | 1 | 0 | 0 | 1 |  |
|  | July | 0 | 0 | 0 | 0 | 0 |  |
|  | August | 0 | 0 | 0 | 0 | 0 |  |
|  | September | 0 | 0 | 0 | 1 | 1 |  |

|  | October | 0 | 0 | 0 | 1 | 1 | 58 |
| --- | --- | --- | --- | --- | --- | --- | --- |
|  | November | 0 | 0 | 0 | 1 | 1 |  |
|  | December | 0 | 0 | 0 | 2 | 2 |  |
|  | January | 2 | 2 | 1 | 9 | 14 |  |
|  | February | 11 | 8 | 0 | 5 | 24 |  |
| **Season 2018/2019** | March | 8 | 6 | 0 | 1 | 15 |  |
|  | April | 1 | 2 | 1 | 1 | 5 |  |
|  | May | 0 | 0 | 0 | 0 | 0 |  |
|  | June | 0 | 0 | 0 | 0 | 0 |  |
|  | July | 1 | 0 | 0 | 0 | 1 |  |
|  | August | 0 | 0 | 0 | 0 | 0 |  |
|  | September | 0 | 0 | 0 | 1 | 1 |  |
|  | October | 0 | 1 | 0 | 0 | 1 | 25 |
|  | November | 0 | 0 | 0 | 0 | 0 |  |
|  | December | 0 | 2 | 0 | 0 | 2 |  |
|  | January | 0 | 1 | 8 | 2 | 11 |  |
|  | February | 1 | 6 | 3 | 0 | 10 |  |
| **Season 2019/2020** | March | 2 | 5 | 2 | 0 | 9 |  |
|  | April | 1 | 4 | 0 | 0 | 5 |  |
|  | May | 1 | 1 | 0 | 0 | 2 |  |
|  | June | 0 | 0 | 0 | 0 | 0 |  |
|  | July | 0 | 0 | 0 | 0 | 0 |  |
|  | August | 0 | 0 | 0 | 0 | 0 |  |
|  | September | 0 | 0 | 0 | 0 | 0 |  |
|  | October | 0 | 0 | 0 | 0 | 0 | 36 |
|  | November | 0 | 0 | 0 | 0 | 0 |  |
|  | December | 0 | 0 | 0 | 0 | 0 |  |
|  | January | 0 | 0 | 0 | 0 | 0 |  |
|  | February | 0 | 0 | 0 | 0 | 0 |  |
| **Season 2020/2021** | March | 0 | 0 | 0 | 1 | 1 |  |
|  | April | 0 | 2 | 0 | 0 | 2 |  |
|  | May | 1 | 1 | 0 | 0 | 2 |  |
|  | June | 1 | 1 | 0 | 1 | 3 |  |
|  | July | 0 | 1 | 0 | 0 | 1 |  |
|  | August | 0 | 0 | 0 | 0 | 0 |  |
|  | September | 0 | 0 | 0 | 0 | 0 |  |
|  | October | 0 | 0 | 0 | 4 | 4 | 28 |
|  | November | 1 | 0 | 0 | 2 | 3 |  |
|  | December | 1 | 0 | 0 | 4 | 5 |  |
|  | January | 1 | 0 | 0 | 4 | 5 |  |
|  | February | 1 | 0 | 0 | 0 | 1 |  |
| **Season 2021/2022** | March | 1 | 0 | 0 | 0 | 1 |  |
|  | April | 0 | 0 | 0 | 0 | 0 |  |
|  | May | 0 | 0 | 1 | 0 | 1 |  |
|  | June | 1 | 0 | 0 | 0 | 1 |  |
|  | July | 0 | 0 | 0 | 0 | 0 |  |
|  | August | 0 | 0 | 0 | 0 | 0 |  |
|  | September | 0 | 0 | 0 | 0 | 0 |  |
| **Total** |  | 105 | 120 | 63 | 181 |  | 469 |

**Figure S1a.**

Monthly distribution of new HCoV-229E cases (*n*=105, bars). The dotted line represents the total amount of detected HCoV.

**Figure S1b.**

Monthly distribution of new HCoV-NL63 cases (*n* =120, bars). The dotted line represents the total amount of detected HCoV.

**Figure S1c.**

Monthly distribution of new HCoV-HKU1 cases (*n* =63, bars). The dotted line represents the total amount of detected HCoV.

**Figure S1d.**

Monthly distribution of new HCoV-OC43 cases (*n* =181, bars). The dotted line represents the total amount of detected HCoV.

**Table S2.** Co-infecting pathogens.

| Bacteria | *n* | Viruses | *n* | Fungi | *n* |
| --- | --- | --- | --- | --- | --- |
| *Staphylococcus aureus* | 18 | Influenza A H1N1 | 10 | *Pneumocystis jiroveci* | 3 |
| *Streptococcus pneumoniae* | 8 | RSV | 12 | *Aspergillus spp.* | 3 |
| *Haemophilus influencae* | 8 | Influenza A H3N2 | 10 |  |  |
| *Pseudomonas aeruginosa* | 5 | Rhinovirus | 10 |  |  |
| *Escherichia coli* | 4 | Parainfluenza type 3 | 6 |  |  |
| *Klebsiella pneumoniae* | 4 | Influenza B | 4 |  |  |
| *Enterococcus faecium* | 2 | Parainfluenza type 4 | 3 |  |  |
| *Moraxella catarrhalis* | 3 | CMV | 3 |  |  |
| *Klebsiella oxytoca* | 3 | Bocavirus | 2 |  |  |
| *Enterobacter cloacae* | 3 | Parainfluenza type 1 | 1 |  |  |
| *Steptococcus dysgalactiae* | 2 | Parainfluenza type 2 | 1 |  |  |
| *Proteus mirabilis* | 2 | Metapneumono Virus | 1 |  |  |
| *Serratia marcescens*  *Serratia marcescens* | 2 | Adenovirus | 1 |  |  |
| *Streptococcus agalactiae* | 1 |  |  |  |  |
| *Granulicatella adiacans* | 1 |  |  |  |  |
| *Raoultella ornithinolytica* | 1 |  |  |  |  |
| *Pseudomonas fluorescens* | 1 |  |  |  |  |
| *Pseudomonas putida* | 1 |  |  |  |  |
| *Acinetobacter Baumannii 100.00CFU/ml* | 1 |  |  |  |  |
| *Acinetobacter pitii* | 1 |  |  |  |  |
| *Mycoplasma pneumoniae* | 1 |  |  |  |  |
| *Stenotrophomonas maltophilia* | 1 |  |  |  |  |
| *Mykobakterium chimaera* | 1 |  |  |  |  |

Pathogens detected by type, with n showing their frequencies of detection. The co-infections included cases with detections of more than one pathogen.

**Table S 3.** Stratification of Co-infecting viral pathogens.

| Co-Infection | HCOV-229E | HCoV-NL63 | HCoV-HKU-1 | HCoV-OC43 |  |
| --- | --- | --- | --- | --- | --- |
| Influenza A | 6 | 5 | 1 | 8 |  |
| RSV | 3 | 5 | 0 | 5 |  |
| Rhinovirus | 2 | 3 | 0 | 4 |  |
| Parainfluenza | 3 | 3 | 3 | 2 |  |
| Influenza B | 1 | 1 | 1 | 1 |  |
| CMV | 0 | 1 | 2 | 0 |  |
| Bocavirus | 0 | 1 | 0 | 1 |  |
| Human metapneumovirus | 1 | 0 | 0 | 0 |  |
| Adenovirus | 1 | 0 | 0 | 0 |  |
|  |  |  |  |  |  |

Pathogens detected by type, with n showing their frequencies of detection. The co-infections included cases with detections of more than one pathogen.

**Table S 4.** Stratification of co-infecting bacterial pathogens.

| Co-Infection | HCOV-229E | HCoV-NL63 | HCoV-HKU-1 | HCoV-OC43 |
| --- | --- | --- | --- | --- |
| *Staphylococcus aureus* | 4 | 4 | 2 | 8 |
| *Streptococcus pneumoniae* | 1 | 3 | 1 | 3 |
| *Haemophilus influencae* | 2 | 2 | 3 | 1 |
| *Pseudomonas aeruginosa* | 0 | 1 | 1 | 3 |
| *Enterobacteriacae* | 5 | 5 | 3 | 8 |
| *Moraxella catarrhalis* | 1 | 0 | 1 | 1 |

Pathogens detected by type, with n showing their frequencies of detection. The co-infections included cases with detections of more than one pathogen.

**Table S 5.** Clinical characteristics and outcomes in patients with co-infection.

|  |  | **Without Co-infection** | **With Co-infection** | ***p*-Value** |
| --- | --- | --- | --- | --- |
| **Clinical presentation** |  |  |  |  |
| Fever | [% (n/total)] | 34.6 (79/228) | 47.9 (45/94) | **0.027** |
| Dyspnea | [% (n/total)] | 31 (76/245) | 52.1 (49/94) | **< 0.001** |
| URTI | [% (n/total)] | 57.7 (131/227) | 42.4 (36/85) | **0.015** |
| LRTI | [% (n/total)] | 27.2 (85/313) | 61.7 (71/115) | **< 0.001** |
| Pneumonia | [% (n/total)] | 16 (50/313) | 48.7 (56/115) | **< 0.001** |
| Bronchitis | [% (n/total)] | 5.4 (17/313) | 11.3 (13/115) | **0.035** |
| Exacerbation of OLD | [% (n/total)] | 10.2 (32/313) | 12.2 (14/115) | n.s. |
| ARDS | [% (n/total)] | 1.8 (6/337) | 8.4 (10/119) | **0.001** |
| ICU stay | [% (n/total)] | 18.3 (62/339) | 35.3 (42/119) | **< 0.001** |
| Ventilation | [% (n/total)] | 12.4 (42/339) | 25,2 (30/119) | **0.001** |
| In-hospital mortality | [% (n/total)] | 7.7 (26/339) | 13.4 (16/119) | n.s. |
| Systemic prednisolone administration | [% (n/total)] | 8.8 (29/328) | 14.5 (17/117) | n.s. |
| Bronchodilator | [% (n/total)] | 12.5 (41/328) | 12.8 (15/117) | n.s. |
| Length of stay [days] | [median (IQR)] | 13 (7-24) | 16 (7.75-27) | n.s. |
| Length of ICU-stay [days] | [median (IQR)] | 4 (2-8.25) | 7 (3.75-14.25 | **0.004** |

Data are presented as frequencies (% [n/total]) or as median (IQR). In the presence of missing data, “(n/total)” indicates the number of cases meeting the respective criterion (n) out of the number of cases with available data for that parameter (total).

**Table S 6.** Distribution of co-infection types according to LRTI status.

| **Co-infection** |  | | **LRTI** | **No LRTI** | ***p*-Value** |
| --- | --- | --- | --- | --- | --- |
| Only viral | | [% (n/total)] | 10.9 (17/156) | 10.7 (29/272) | n.s. |
| Only bacterial | | [% (n/total)] | 22.4 (35/156) | 4.4 (12/272) | **<0.001** |
| Only fungal | | [% (n/total)] | 3.8 (6/156) | 0.7 (2/272) | **0,03** |
| Mixed | | [% (n/total)] | 8.3 (13/156) | 0.4 (1/272) | **<0.001** |

Pathogens detected by type presented as frequencies (% [n/total]). The co-infections included cases with detections of more than one pathogen.
